# A matched group study of triple negative versus HER-2 positive (irrespective of hormonal status) breast cancer: two subtypes with high-risk features and poor outcome

**DOI:** 10.3332/ecancer.2010.167

**Published:** 2010-08-27

**Authors:** J M Zekri, E Ibrahim, B Ben Sadiq, AM Al-Gahmi, AA Zeeneldin, TR Elkhodary, HE Gaballa, EE Fawzy, ME Elsayed, Y Bahadur, S Awadalla, MS Alzahran

**Affiliations:** 1 Department of Oncology; 2 Research Centre, King Faisal Specialist Hospital & Research Centre, Jeddah 21499, Kingdom of Saudi Arabia

## Abstract

**Introduction:**

Genetic profile studies of breast cancer identified a number of biologically different subtypes. These genetic subtypes are often surrogated by oestrogen receptors (ERs), progesterone receptors (PR) and HER2 status as measured by immunohistochemistry (IHC). Triple negative (TN) subtype is recognized to have high-risk features and poor outcome. Over-expression of the HER2 is also recognized as poor outcome marker. The characteristics and outcome of HER2 positive tumours (irrespective of hormonal status) (HER2 HR+/−) identified by IHC have not addressed in the era of surrogate genetic subtyping. Therefore, we retrospectively compared the risk features and clinical outcome of patients with TN against these with HER2 HR+/− tumours.

**Patients and Methods:**

Forty patients with HER2 HR+/− tumours were matched for age and stage to 40 patients with TN tumours. Clinical and pathological data were collected retrospectively. All patients were managed in a single institution.

**Results:**

Tumour grade and stage and rate of pathologically involved lymph nodes were similar in both groups. There was a trend of more lymphovascular invasion in HER2 HR+/− than TN patients (40% vs. 27.5%. p=0.07). Relapse and death rates were not statistically different (p=0.469 and p=1.0, respectively). Median relapse free survival was 38 months for TN and not reached for HER2 HR+/− patients (Log rank; p=0.757). Median overall survival was not reached in both groups. Multivariate analysis did not identify TN or HER2 HR+/− status to have any differential impact on RFS.

**Conclusion:**

HER2 HR+/− tumours exhibit high risk, presenting features and relatively poor clinical outcome possibly not very different from the increasingly recognized TN tumour.

## Introduction

The class discovery expression profile studies pioneered by the Stanford group [1, 2] have demonstrated that the morphological heterogeneity of breast cancer can be recapitulated and systematically classified at the transcriptomic level. These studies have shown that the expression profiles of breast cancer display a systematic variation and allow classification of breast cancer into five main groups, two of them ER+ (oestrogen receptor positive)[luminal A and B] and three ER− (oestrogen receptor negative) groups [normal breast-like, ERBB2 (also known as HER2) and ‘basal-like’][1, 3].

In these, and in subsequent studies, it has been shown that the basal-like group is enriched for tumours that lack expression of hormone receptors and of HER2 and has a more aggressive clinical behaviour, a distinctive metastatic pattern [4, 5] and a poor prognosis despite responding to conventional neoadjuvant and adjuvant chemotherapy regimens[6, 7].

The basal-like subtype accounts for about 15% of breast cancer cases. Because basal-like breast cancers are ER−, PR− (progesterone receptor negative) and HER2 negative, they are sometimes called ‘triple negative’, (TN). Some investigators have concluded that TN breast cancer is synonymous with basal-like breast cancer although it should be noted that only about 85% of triple-negative phenotypic breast cancers are deemed to be basal-like when tested by appropriate immunohistochemical means [8].

Over-expression of the HER 2 oncoprotein irrespective of hormone receptor status (HER2 HR+/−) is a well-known adverse prognostic factor associated with poor relapse free (RFS) and over all survival (OS) in breast cancer [9].

A number of studies investigated the characteristics of TN tumours in comparison with a cohort of mix of other subtypes [10, 11]. However, there are no data in the literature comparing TN and HER2 HR+/− groups, two subtypes with seemingly poorest outcome.

Gene expression analysis has not become a standard test in daily clinical management of breast cancer. However, physicians are increasingly using ER, PR and HER2 status to try and predict tumour behaviour and clinical outcome. The five identified subtypes based on receptors status do not include a category of HER2 HR+/−. About 20%–25% of patients fall in this group. In this report, we investigate the clinical characteristics and outcome of HER2 HR+/− and compare it to that of age and stage matched TN patients in a cohort of patients with early breast cancer.

## Patients and methods

### Patients and data source

Forty patients with HER2 HR+/− tumours were identified. 1:1 matching for age and stage was performed to identify 40 patients with TN tumours. All patients attended King Faisal Specialist Hospital and Research Centre-Jeddah, Kingdom of Saudi Arabia. Patients were identified from the hospital oncology electronic database.

All patients received their management at our facility from January 2002 to December 2007. Initial evaluation included clinical examination, mammography and breast ultrasound. Computed tomography of chest, bone scan and breast magnetic resonance imaging were performed if indicated. Clinical data were collected from the oncology database, electronic results system and supplemented by retrospective review of patients’ medical records. The hospital’s institutional review board (ethical committee) approved the project.

## Immunohistochemistry analysis

Immunohistochemical staining was carried out using standard streptavidin-biotin peroxidase method on 3- to 5-mm-thick tissue sections. Staining was performed with antibodies raised against the following markers: ER, PR and HER 2. ER and PR status were recorded according to the pathologist’s interpretation of the assays. ER and PR were considered negative if immunoperoxidase staining of tumour cell nuclei is <5%. A negative HER-2 expression using HercepTest (Dako, Glostrup, Denmark) was defined as no membranous staining (negative) or those that either had some staining in <10% of tumour cells or had weak-to-moderate staining (1+). Those who had moderate staining in >10% of cells (2+) were further evaluated by fluorescence *in situ* hybridisation (FISH) for gene amplification. FISH is scored on a quantitative scale with less than two copies of the HER-2 gene classified as negative.

## Statistical analysis

Demographic, clinical, pathological and treatment variables in both groups were compared using Chi-squared test for categorical variables. T test was used to compare means. RFS was calculated from date of diagnosis to date of relapse or last follow-up. OS was calculated from date of diagnosis to date of death or last follow-up. RFS and OS were computed using survival tables and Kaplan Meier method. Log rank test was used to test the difference between both groups. Cox regression model was used to calculate hazards ratio and to adjust for potential prognostic variables

## Results

Table 1 illustrates patients’ characteristics and treatment for the whole cohort and for each group. Patients’ age and tumour stage in both groups were identical due to the matched design of the study. Tumour grade was similar in both groups. LVI was reported more often in HER2 HR+/− than TN patients (40% vs. 27.5%, p=0.07). Fifty percent of HER2 HR+/− patients were ER+, 45% were PR+, 45% were positive for both receptors and 50% were HR+ (positive ER and/or PR). Treatments (type of surgery, neoadjuvant chemotherapy, adjuvant chemotherapy and adjuvant radiotherapy) were similar in both groups. The difference in hormone receptor status and adjuvant hormonal therapies reflects the nature of patients in the study.

After a median follow-up of 21.5 months, 35% and 27% relapsed and 7.5% and 7.5% died in TN and HER2 HR+/− groups, respectively (p=not significant) (Table 2). Median RFS was 38 months for TN and not reached for HER2 HR+/− patients (p=0.043). Median OS was not reached in both groups (Table 2).

**Figure 1:**
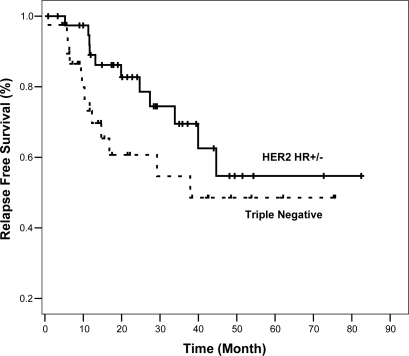
Relapse free survival of TN and HER2 HR+/− groups (*n*=80).

**Figure 2:**
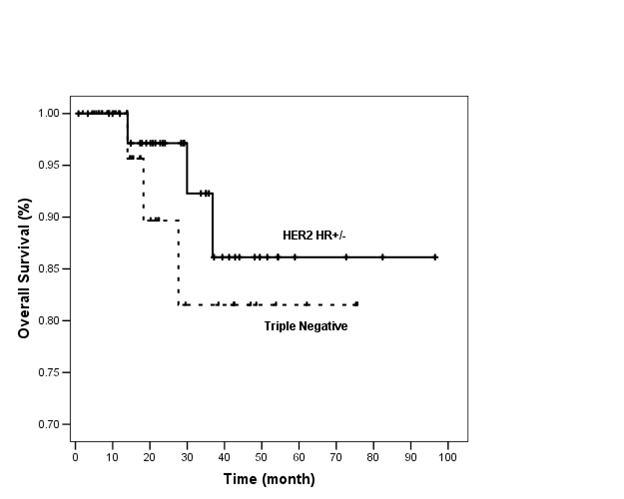
Overall survival of TN and HER2 HR+/− groups (*n*=80).

At 3 years, RFS was 55% and 70% and OS was 81% and 93% in TN and HER2 HR+/− groups, respectively (Figures 1 and 2).

In HER2 HR+/− group, 20 patients had HR+ disease, 6/20 (30%) relapsed. Twenty patients had HR− disease, 5/20 (25%) relapsed. The difference in relapse rate between HR+ and HR− subgroups was not statistically significant (p=0.723). The median RFS was not reached in these subgroups (p=0.757).

On Cox regression multivariate analysis, stage, number of pathologically involved lymph nodes, grade, TN and HER2 HR+/− were found to have no significance impact on RFS.

## Discussion

Tumours that over-express HER-2 can be ER− or ER+. Oncologists are well aware that HER-2 over-expression is an independent poor prognostic factor (9). The incorporation of Trastuzumab in the adjuvant and palliative treatments improved the outcome [12, 13].

The TN tumours lack the expression of all three routinely tested receptors (ER, PR and HER-2) and are being increasingly recognized among oncologists as another poor prognostic group.

Both TN and HER2 HR+/− tumours present with high-risk features that include higher stage, high-pathological grade and involvement of lymph nodes, and at the same time both share a poor outcome.

In this report, we study the tumour features and outcome of these two seemingly poorest outcome groups after matching for two independent prognostic factors, that is age and stage.

The mean age of our patients is relatively young 45 (42–49) years. It is possible that part of this is artificial due to matching process. However, other factors may explain this finding; (I) patients with TN tumours present at a younger age than other subtypes [11], (II) the frequency of HER-2 over-expression decreases with increasing age at diagnosis [14] although this may not be a unanimous finding as some data show HER-2 over-expression not significantly different in women <35 and >=35 years [15], (III) women in Saudi Arabia present with breast cancer at younger age. In the 2004 Saudi cancer incidence report, 44% and 59% of patients were <45 and <50 years, respectively (16). In a retrospective review of 780 patients who received chemotherapy in Saudi Arabia, 64% of patients were <50 years and 62% were pre-menopausal [3].

HER-2 tumours are more likely to be ER− or express lower levels of ER than if they are Her-2 negative [14, 17]. However, the association between ER, PR and HER-2 over-expression varies with age. The hormone receptors are not an independent predictor for HER-2 expression in young women while they are in elder patients (>45 years) (18). Fifty percent of our patients were HR+. This is generally accepted representation for this group of patients. In the HERA phase III international study, 50.8% and 50.1% of HER-2 tumours were HR+ in the 1-year trastuzumab and the observation arms, respectively. This suggests that despite small sample size and selection for matching our sample represents the true characteristics and features of this group of tumours [12]. The mean age of our patients was 45 years, and all were younger than 50 years. This may have had an impact on the loss of HR-predominance.

More than half of patients in each group had pathologically involved lymph nodes (TN: 61.5% and HER2 HR+/−: 53.8% P 0.492). This high-risk feature is present and not statistically different in both groups of our patients. There are conflicting results on the prevalence of lymph node metastasis at the time of diagnosis in patients with TN cancers, whereas in one study there was a higher prevalence of lymph node metastasis in TN compared with non-TN controls (54.4% vs. 45.6% p=0.02) [11]; others have found an opposite association (24.1% vs. 42.1% p=0.01) (19). It is worth mentioning that the controls in these two comparisons were unselected patients with breast cancer that contained a mix of all subtypes. The lack of significant difference in rate of involved lymph nodes in our patients indicates that both groups likely share high-risk features. However, this may be at least partly explained by the design of matching for stage. 87.5% of our patients in each group had stage II or III. This mostly indicates N1 or N2 in TNM classification (although some patients with stage II can be N0 in AJCC staging). In a closer look at the numbers of involved lymph nodes, we found that in TN and HER2 HR+/−, 8/39 (20.5%) and 17/39 (43.6%) had four or more involved lymph nodes while 16/39 (40%) and 4/39 (10.3%) had one to three involved lymph nodes, respectively. This suggests that HER2 HR+/− may present with higher risk feature than TN when considering number of involved lymph nodes.

Lymphovascular invasion (LVI) and high pathological grade are features frequently reported in TN tumours. In a previous work our group compared features of TN and Luminal A tumours. The group found more LVI and grade III features in TN than in luminal A tumours (p=0.03 and 0.007), respectively. These findings were also reported by a Canadian group comparing TN and other pathological types in a cohort of 1601 patients [11]. In the current study, we found a trend of more LVI in HER2 HR+/− than in TN; 40% and 27.5% (p=0.07). This did not reach statistical significance probably due to relatively small sample size and lack of LVI data in 32.5% and 22.5% of patients. Pathological Grade III was similar in both groups. This points to the possibility that HER2 HR+/− also present with poor prognostic factors similar to or may be worse than TN tumours.

Despite shorter median duration of follow-up for the TN group (15 vs. 29.6 months), the rate of relapse was 35% in the TN group and 27.5% in the HER2 HR+/− (p=0.469). It is logical to assume more relapses will have occurred if TN group were followed up longer. However, it is possible that longer follow-up may not yield significantly more relapses as it is well documented that peak risk of recurrence in TN tumours occurs relatively early between the first and third years [11, 19]. This phenomenon of early recurrence with in 3 years is also supported by our finding of the inferior 3-year RFS of TN group compared to HER2 HR+/− (55% vs. not reached; p=0.043).

Adjuvant trastuzumab became a standard adjuvant therapy for women with intermediate and high-risk HER2 breast cancer tumours in December 2005 at our institution. For this reason, only 15/40 (37.5%) patients received adjuvant trastuzumab. The other 25 patients did not receive adjuvant trastuzumab either due to diagnosis before this era, presence of medical contraindication or lack of other intermediate and/or high-risk features. It is reasonable to suggest that in this day and time more patients will receive adjuvant trastuzumab, and this will impact favourably at PFS for this group.

On multivariate analysis, there was no significance impact on TN and HER2 HR+/− status on RFS, suggesting that these two factors per se impose similar outcome.

There were only three deaths in each group. Three year OS was 81% and 93% in TN and HER2 HR+/− groups, respectively (p=0.35). The lack of difference in survival can be due to: (I) short duration of follow-up. (II) Patients in TN group who relapsed toward the end of 3 years may have had good response to salvage chemotherapy and thus did not translate to early death. It is well reported that TN tumours respond well to neoadjuvant chemotherapy [6]. However, data on outcome in response to chemotherapy after recurrence are sparse and there remains considerable heterogeneity in individual outcomes [20]. (III) Probable true similar survival outcome in these two groups, namely TN and HER2 HR+/−. (IV) Only 37.5% of HER2 HR+/− patients received adjuvant trastuzumab.

More accurate information on recurrence and mortality will be obtained with longer follow-up of our cohort and with multi-institutional approach to study larger number of patients. Targeting patients treated in the era of adjuvant trastuzumab in future studies will make results more clinically relevant to current practice.

It is worth reminding here that there are other subtypes of breast cancer identified on genetic profiling. The luminal subtype C is further distinguished from luminal subtypes A and B by the high expression of a novel set of genes whose coordinated function is unknown, which is a feature they share with the basal-like and ERBB2 subtypes [1]. The luminal subtype B and C tumours might represent a clinically distinct group with a different and poor disease course, in particular with respect to relapse. Luminal subtype C is associated with the worst outcome of the three luminal subtypes. The potential clinical significance of this molecular subtype is highlighted by the similarities in expression of some of the genes that are characteristic of the ER-tumours in the basal-like and ERBB2 subtypes, which suggests that the high level of expression of this set of genes is associated with poor disease outcomes. Based on the above work by Sørlie T *et al* [1], it is possible that proportion of HER2 HR+/− patients in our series belong to the luminal C subtype. This may explain their high-risk features at presentation and a relatively high overall relapse rate (27.5%). Further genetic profile work is needed to classify HER2 HR+/− tumours.

## Conclusion

At the current era of surrogacy of genetic profile and routine pathological feature of breast cancer, we identify HER2 HR+/− tumours as a subtype with high risk, presenting features and relatively poor clinical outcome possibly not very different from the increasingly recognized TN tumours.

## Figures and Tables

**Table 1:**
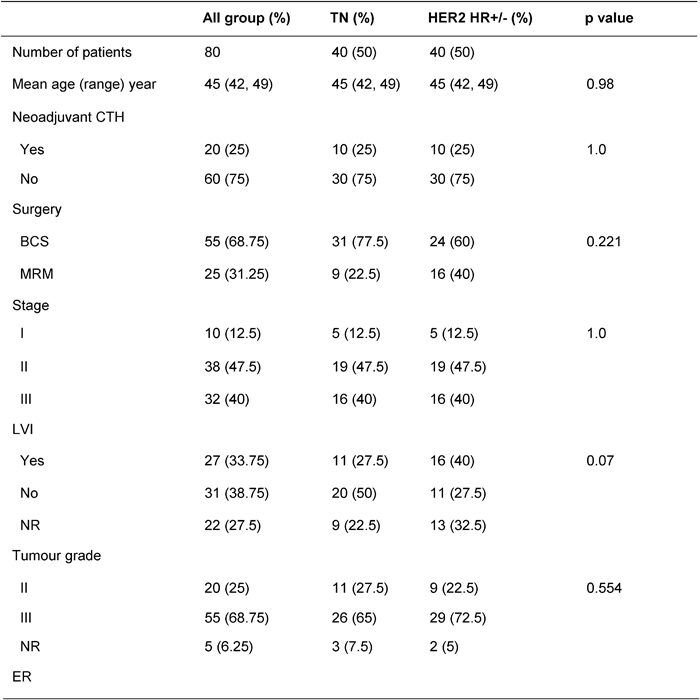

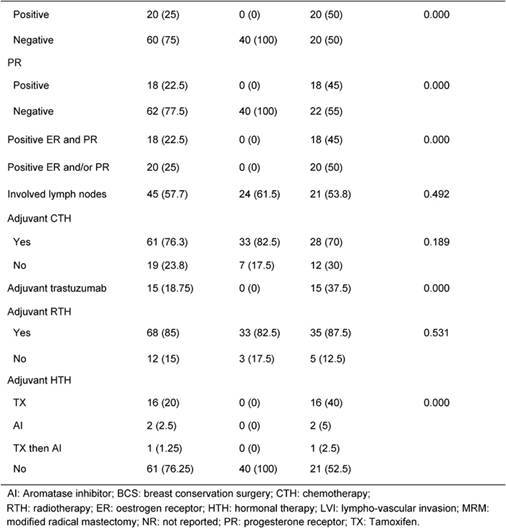
Patients’ characteristics and treatment

**Table 2:**
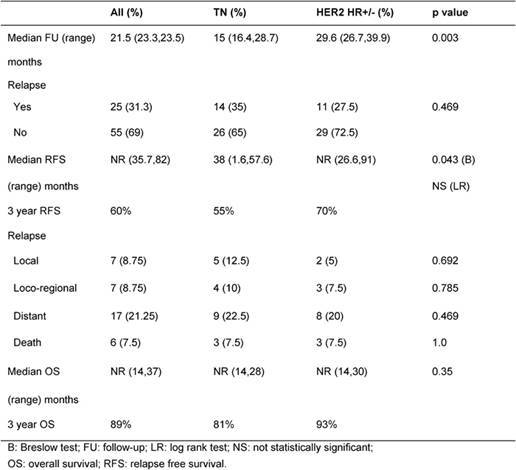
Breast cancer outcome: Rates of relapse and death
